# Editorial: Challenges and solutions for the incomplete immune restoration in HIV-infected patients under antiretroviral therapy

**DOI:** 10.3389/fcimb.2023.1232699

**Published:** 2023-06-14

**Authors:** Yi-Qun Kuang, Caijun Sun, Daniel R. Ram, Christiane Moog, Bin Su

**Affiliations:** ^1^ Research Center for Clinical Medicine, First Affiliated Hospital of Kunming Medical University, Kunming Medical University, Kunming, Yunnan, China; ^2^ School of Public Health (Shenzhen), Sun Yat-sen University, Guangdong, China; ^3^ Burnett School of Biomedical Sciences, College of Medicine, University of Central Florida, Orlando, FL, United States; ^4^ Laboratoire d’ImmunoRhumatologie Moléculaire, INSERM UMR_S 1109, Fédération de Médecine Translationnelle de Strasbourg (FMTS), Université de Strasbourg, Strasbourg, France; ^5^ Beijing Key Laboratory for HIV/AIDS Research, Sino-French Joint Laboratory for Research on Humoral Immune Response to HIV Infection, Clinical and Research Center for Infectious Diseases, Beijing Youan Hospital, Capital Medical University, Beijing, China

**Keywords:** HIV, immunological non-responders, Immune restoration/reconstitution, antiretroviral therapy, CD4^+^ T cells

The latest estimates from the Joint United Nations Programme on HIV/AIDS (UNAIDS) show that the world continues to close in on the goal of ending the AIDS epidemic by stopping HIV transmission and reducing AIDS-related deaths, but approximately 38.4 million people globally were still living with HIV in 2021 (Unaids, 2021). Antiretroviral therapy (ART) has been proven effective at reducing viral loads to undetectable levels, reducing the morbidity and mortality of AIDS-related diseases, and recovering CD4^+^ T cells to restore the immune functions of HIV-infected patients (Ghosn et al., 2018). However, approximately 10%-40% of HIV-infected individuals fail to achieve normalization of CD4^+^ T cells above 500 cells/µL despite effective virological suppression with ART (Yang et al., 2020; Su et al., 2022). These patients are called immune non-responders (INRs) and show severe immune dysfunction. The incidence and mortality of AIDS-related and non-AIDS-related diseases such as liver disease, cardiovascular disease, non-AIDS-related tumors, and HIV-related neurocognitive impairment were significantly higher in INR patients than in HIV-infected patients who achieved complete immune reconstitution (Battegay et al., 2006; Corbeau and Reynes, 2011).

Although researchers have made various attempts to improve the level of immune reconstitution in INR patients, these specific interventions have not yet achieved convincing results except for standard ART regimens, mainly because the underlying mechanism of incomplete immune restoration in HIV-infected patients has not been fully elucidated. The decreased hematopoiesis of bone marrow, insufficient thymic output, residual viral replication, aberrant immune activation, microbial translocation, immunosenescence, and specific genetic or metabolic characteristics, etc., may impair immune reconstitution in HIV-infected patients (Yang et al., 2020; Su et al., 2022). None of the abovementioned independent factors can fully explain the mechanism of incomplete immune restoration. Therefore, in view of this limitation, in this Research Topic, we invited articles that further our understanding of the immune restoration mechanism and the consequences thereof. This Research Topic contains five original research articles and one brief research report that address the mechanisms, challenges, and solutions of incomplete immune restoration in HIV-infected patients under ART.

The research article by Li et al. investigated trends in clinical monitoring indices in HIV/AIDS patients after 9.9 years of ART in Yunnan Province, China. Three machine learning models (support vector machine [SVM], random forest [RF], and multi-layer perceptron [MLP]) were constructed. They used clinical indicators such as routine blood examination, lymphocyte subset counts, and viral load as parameters to predict immune restoration. Baseline and follow-up results of routine blood and organ function tests were used to analyze and predict CD4^+^ T-cell data after treatment during long-term follow-up. The authors found that the predictive capability of the three models was better for patients with a CD4 T-cell count ≥ 200 cells/μl than for those with < 200 cells/μl. For both groups, the three models yielded the best predictive performance for the CD4/CD8 ratio, for which the results were highly consistent. In patients with a CD4 T-cell count of < 200 cells/μl, the SVM model exhibited the best performance for predicting the CD4/CD8 ratio, while in patients with a CD4 T-cell count of ≥ 200 cells/μl, the RF model was best. Therefore, by the incorporation of clinical indicators in SVM, RF, and MLP machine learning models, the immune function and recuperation of HIV/AIDS patients can be predicted and evaluated, thereby better guiding clinical treatment.

The brief research report article by Sisteré-Oró et al. described SARS-CoV-2-specific antibody and cellular immune responses in a small cohort of HIV-INR patients after receiving the COVID-19 mRNA-based BioNTech/Pfizer vaccine. Compared to the control group of vaccinated healthy individuals who all developed a virus-specific immune response, 5 of 10 vaccinated HIV-INR patients showed insufficient immune responses. The lack of response was not directly correlated with patient CD4 T-cell counts. Three of the five non-responders who agreed to receive a booster vaccination subsequently generated a virus-specific response. Thus, even HIV-INR patients can be efficiently vaccinated against SARS-CoV-2 infection but may require follow-up by virus-specific immune monitoring to guarantee clinical vaccine benefits. This study demonstrates a significant impairment in approximately 50% of HIV-INR patients of adequately generating SARS-CoV-2-specific immune responses after vaccination. Thus, to provide optimal SARS-CoV-2-preventive health care for this vulnerable patient group, the level of vaccine-induced immune responses should be followed by diagnostic assays, and booster vaccination should be offered if antibody levels are low.

Natural killer (NK) cells play a crucial role in controlling HIV replication, with a potential downstream impact on the size of the HIV reservoir and viral rebound after ART cessation. It is important to understand how primary HIV infection (PHI) disrupts NK cell function and how these functions are restored by early ART. The research article by Pace et al. investigated the impact of commencing ART during PHI on phenotypic and functional NK cell markers at treatment initiation (baseline), 3 months, 1 year, and 2 years in seven well-characterized participants in comparison to HIV seronegative volunteers. Authors then examined how those NK cell properties differentially impacted by ART related to time of viral rebound and HIV DNA levels in 44 individuals from the SPARTAC trial. In this trial, ART started during PHI was stopped after 48 weeks of treatment. This work indicates that early ART started in PHI incompletely restores the NK cell phenotype and function imposed by HIV infection. While there is evidence that NK cells play a role in delaying viral rebound and limiting HIV DNA levels, no single NK cell marker defined was associated with delayed viral rebound. This study provides a rationale for targeting NK cells in HIV cure strategies.

To improve the survival of HIV/AIDS patients on ART, information on factors associated with the time to ART adverse drug reactions and their predictors is needed. However, there is a paucity of evidence on the time for ART adverse drug reactions and its predictors among HIV/AIDS patients in Ethiopia. The research article by Weldesenbet et al. investigated the time to first ART adverse drug reaction and its predictors among adult HIV/AIDS patients on first-line ART in the West Hararghe Zone, Eastern Ethiopia, from 2013–2019. The authors showed that most of the ART adverse drug reactions occurred within one year of ART initiation. The initial ART regimen (TDF, 3TC, EFV), adherence, HIV/AIDS stage, and body mass index (BMI) were risk factors for the time to ART adverse drug reaction. The incidence of ART adverse reactions was relatively low with early onset treatments. Patients with poor adherence need to receive continuous counseling to improve their adherence status. This finding will be helpful for the timely identification and treatment of adverse drug reactions caused by ART.

Liu et al. conducted a prospective cohort study to explore the potential of lenalidomide as an immunomodulatory agent in HIV-1-infected patients. The initial goal of this study was to assess whether lenalidomide improves chronic intracranial inflammatory injury in HIV-associated cryptococcal meningitis (HIV-CM) through 24-week treatment. Somewhat by accident, the authors found that lenalidomide not only improved persistent central inflammatory injury but also inhibited proinflammatory cytokine secretion and reduced cell-associated (CA) HIV RNA levels. Therefore, they showed for the first time that lenalidomide reduces the level of CA HIV RNA and improves persistent inflammation in patients with HIV-CM. They propose that lenalidomide, may be used in the clinic as a potential inhibitor of viral transcription in HIV-1 reservoirs and reduce HIV-related inflammation.

The rising levels of HIV drug resistance have significantly hampered the anticipated success of ART in HIV-1-infected individuals, particularly those from Africa. The research article by Parbie et al. investigated patterns of drug resistance mutations after first-line regimen ART in patients from Ghana. Blood samples were collected from HIV-1-infected individuals (≥18 years) attending the HIV/AIDS clinic at the Eastern Regional Hospital, Koforidua, Ghana from September to October 2017. In a cross-sectional study, they compared naïve as well as ART-experienced HIV-1-infected individuals. They reported high-level resistance to non-nucleoside reverse transcriptase inhibitor (NNRTI)-based first-line regimens in Ghana and provided evidence of the effectiveness of protease inhibitors (PI) and integrase strand transfer inhibitors (INSTI) in Ghana since no major drug resistance mutations were detected against these drugs. Thus, the absence of major PI- and INSTI-associated mutations is a good signal that the current WHO recommendation of Dolutegravir in combination with a nucleoside reverse transcriptase inhibitor (NRTI) backbone. This combination yielded maximum benefits as a first-line regimen for HIV-1-infected individuals in Ghana. Additionally, they emphasized the importance of overcoming logistics challenges and implementing routine viral load monitoring nationwide.

In conclusion, the six published articles on this Research Topic have advanced our understanding of the interaction between HIV infection and host immune responses, thus improving our understanding of the mechanisms involved. The collection of articles has summarized the latest research in the field and highlighted the challenges and solutions of incomplete immune restoration in HIV-1-infected patients under ART. A better understanding of the nature of the immune reconstitution that results from potent ART is critical to the optimal clinical management of HIV-1-infected individuals. It may provide important insights into the immunopathogenesis of HIV infection as well. This knowledge is essential for the further development of new treatment strategies to improve immune restoration in HIV-1-infected patients.

## Author contributions

Y-QK, and BS conceived this work and drafted the manuscript; Y-QK, and CS revised the draft; DR, and CM made substantial contributions to the work through in-depth discussion. All the authors proposed the Research Topic theme, made a direct and intellectual contribution to the work, and approved the final version for publication.
